# Two Distinct Functional Patterns of Hepatitis C Virus (HCV)-Specific T Cell Responses in Seronegative, Aviremic Patients

**DOI:** 10.1371/journal.pone.0062319

**Published:** 2013-04-30

**Authors:** Yoon Seok Choi, Jung Eun Lee, Seung Joo Nam, Jung Tak Park, Hyon-Suk Kim, Kyu Hun Choi, Beom Seok Kim, Eui-Cheol Shin

**Affiliations:** 1 Laboratory of Immunology and Infectious Diseases, Graduate School of Medical Science and Engineering, KAIST, Daejeon, Republic of Korea; 2 Department of Internal Medicine, Chungnam National University College of Medicine, Daejeon, Republic of Korea; 3 Department of Internal Medicine, Yonsei University College of Medicine, Seoul, Republic of Korea; 4 Department of Laboratory Medicine, Yonsei University College of Medicine, Seoul, Republic of Korea; Mayo Clinic, United States of America

## Abstract

In hepatitis C Virus (HCV) high-risk groups, HCV-specific T cell responses have been detected in seronegative, aviremic persons who have no evidence of HCV infection. Herein, we investigated functional profiles of HCV-specific T-cell responses in seronegative, aviremic patients of a HCV high-risk group. Seventy seven hemodialysis patients with chronic renal disease were analyzed by IFN-γ ELISpot assays, and eight of 71 (11.3%) seronegative, aviremic patients displayed HCV-specific T-cell responses. Their HCV-specific memory T cells were characterized by assessing cytokine polyfunctionality, known to provide antiviral protection. By intracellular staining of IFN-γ, TNF-α, IL-2 and MIP-1β, we identified two distinct populations in the seronegative, aviremic patients: polyfunctional responders and TNF-α-predominant responders. In further analysis, occult HCV infection was excluded as a cause of the HCV-specific T cell response via secondary nested RT-PCR of HCV RNA in peripheral blood mononuclear cell samples. HCV-specific T cells targeted multiple epitopes including non-structural proteins in a single patient, implying that their T cells might have been primed by HCV proteins synthesized within the host. We conclude that HCV-specific memory T cells of seronegative, aviremic patients arise from authentic HCV replication in the host, but not from current occult HCV infection. By functional pattern of HCV-specific T cells, there are two distinct populations in these patients: polyfunctional responders and TNF-α-predominant responders.

## Introduction

Hepatitis C virus (HCV) infection is a major cause of chronic viral hepatitis that often progresses to liver cirrhosis and hepatocellular carcinoma [1]. HCV is an enveloped positive-stranded RNA virus of *Flaviviridae* transmitted by parenteral routes [2]. Persons with frequent exposure to blood, blood products, and contaminated needles are at threat of HCV infection [3]. In fact, healthcare workers, hemophiliacs, hemodialysis patients, and injection drug users (IDUs) are all HCV high-risk groups.

HCV infection is determined clinically by the detection of both anti-HCV antibody and HCV RNA. Seropositivity without viremia is considered a sign of past HCV infection. HCV-specific memory T cells have been detected in persons who spontaneously recovered from past HCV infection [4]–[6]. Intriguingly, HCV-specific T cell responses have been detected not only in persons with past HCV infection but also in seronegative, aviremic persons who have no evidence of current or past HCV infection [7]–[13]. These studies detected T cells reactive to HCV antigens by secreting cytokines such as IFN-γ ELISpot assay or intracellular cytokine staining. HCV-specific T cell responses in seronegative, aviremic persons have been detected mainly in HCV high-risk groups such as IDUs, residents of HCV-endemic areas, healthy family members of HCV-infected patients, and healthcare workers [7]–[13]. However, it remains unclear why HCV-specific T cells are primed in seronegative, aviremic persons. In addition, detailed characteristics of HCV-specific T cells have not been previously identified in seronegative, aviremic persons.

HCV-specific T cell responses in seronegative, aviremic persons can be attributed to several possible causes including occult HCV infection with extremely low level viral replication [14], [15], heterologous T cell immunity by a cross-reactive epitope [16]–[18], transient viral replication without seroconversion [19], [20] and disappearance of anti-HCV antibody long after prior HCV infection [21].

T cells play a major role in the immune response against HCV. In particular, T cell-mediated protective immunity has been shown in chimpanzees that had recovered from previous HCV infection [22], [23]. In these studies, HCV rechallenge resulted in rapid viral clearance with low peak viremia and attenuated liver injury [22]–[24], and this host protection was mediated by memory T cells as evidenced by T cell-depletion studies [25], [26]. Upon HCV rechallenge, depletion of CD4^+^ T cells resulted in chronic persistent infection [25], while depletion of CD8^+^ T cells delayed viral clearance [26].

Recently, T cell-mediated host protection has been shown to be associated with polyfunctional T cells, which exert several effector functions simultaneously [27]–[29]. For example, polyfunctional HIV-specific CD8^+^ T cells were maintained in human immunodeficiency virus (HIV) long-term nonprogressors [28]. In one vaccination study, Th1 cell polyfunctionality correlated with vaccine efficacy, in terms of protection against Leishmania [27]. Therefore, induction of polyfunctional memory T cells is considered a practical goal in T cell vaccine development.

In the present study, we analyzed HCV-specific T cell responses in seronegative, aviremic hemodialysis patients and tried to reveal possible causes of HCV-specific memory T cell induction. We also characterized HCV-specific memory T cells in detail by assessing T cell polyfunctionality, and demonstrated that HCV-specific T cell responders in seronegative, aviremic hemodialysis patients are classified into two distinct groups, polyfunctional responders and TNF-α-predominant responders.

## Materials and Methods

### Ethics Statement

All study samples were obtained following acquisition of the study participants' and/or their legal guardians' written informed consent, in accordance with the Declaration of Helsinki. This research protocol was reviewed and approved by the institutional review board of Yonsei University Severance Hospital.

### Hemodialysis patients

Seventy-seven patients (48 males and 29 females), who were on maintenance hemodialysis due to chronic kidney disease, were recruited in this study. At the time of enrollment, the mean age (±SD) of patients was 57.0±10.6 years, the mean alanine aminotransferase (ALT) level was 17.3±11.6 U/L, and the median duration of hemodialysis was 39.2 months. All cases were negative for anti-HIV. In sera, anti-HCV antibodies were assayed using Elecsys Anti-HCV Immunoassay (Roche Diagnostics, Basel, Switzerland). HCV RNA was quantitated by COBAS TaqMan HCV assay (Roche Diagnostics). The lower detection limit of HCV RNA assay was 15 IU/mL. Peripheral blood mononuclear cells (PBMCs) were isolated from whole blood by standard Ficoll-Paque (GE Healthcare, Uppsala, Sweden) density gradient centrifugation and cryopreserved. In the case of seronegative, aviremic patients with HCV-specific T cell responses, whole blood was obtained a second time to confirm the T cell responses.

### HCV overlapping peptides

Six hundred pentadecamer peptides (Mimotopes, Clayton, Australia), overlapping by 10 amino acids and spanning the complete HCV genotype 1a (H77) sequence, were resuspended at 20 mg/mL in dimethyl sulfoxide (DMSO). Aliquots of each peptide suspension were pooled and diluted with phosphate-buffered saline to obtain 49 mixes designed for a matrix array [30]. Each mix contained 24–25 peptides, and each peptide was present in 2 mixes (Figure S1A). These 49 mixes were used in direct *ex vivo* IFN-γ enzyme-linked immunosorbent spot (ELISpot) assays in each patient.

### Direct ex vivo IFN-γ ELISpot assay

Direct *ex vivo* IFN-γ ELISpot assays were performed as previously described [31]. Duplicate cultures of 250,000 PBMCs were set up in RPMI 1640 containing 5% fetal bovine serum (FBS) and 2 mM L-glutamine with 49 peptide mixes (each peptide at a final concentration of 1 µg/ml). DMSO was used as a negative control. After 30 hours in culture, IFN-γ spots were developed. Spots were counted with an ELISpot reader (CTL, Cleveland, OH), and the number of specific spots was calculated by subtracting the mean number of spots in negative control wells of each ELISpot plate from the mean number of spots in each well stimulated with an HCV peptide mix.

### Intracellular cytokine staining (ICS) and T cell polyfunctionality assay

Cryopreserved PBMCs were thawed, resuspended in RPMI 1640 containing 5% FBS and 2 mM L-glutamine, and rested overnight at 37°C. PBMCs were stimulated with an epitope peptide or a peptide mix (each peptide at a final concentration of 1 µg/ml) in the presence of anti-CD28 and anti-CD49d (1 µg/ml for each; BD Biosciences, San Jose, CA), and brefeldin A (GolgiPlug, BD Biosciences) and monensin (GolgiStop, BD Biosciences) were added 1 hour later. After another 5 hours of incubation, PBMCs were stained with anti-CD3- V500, anti-CD4- V450 and anti-CD8-APC-H7 (all from BD Biosciences), permeabilized using Cytofix/Cytoperm kit (BD Biosciences) and further stained with either anti-IFN-γ-APC or anti-TNF-α-PE-Cy7 (all from BD Biosciences). In the assay for T cell polyfunctionality, anti-CD107a-PE (BD Biosciences) was included in the initial culture medium and PBMCs were stained with anti-IFN-γ-APC, anti-TNF-α-PE-Cy7, anti-MIP-1β-PerCP-Cy5.5 (all from BD Biosciences) and anti-IL-2-Alexa Fluor 488 (BioLegend, San Diego, CA) after permeabilization. FACS analysis was performed by LSRII flow cytometer (BD Biosciences) and the data were analyzed using FlowJo software (Treestar, San Carlos, CA). T cells positive for the various combinations of cytokines and degranulation were quantified and analyzed using a Boolean gating function in FlowJo software.

### Multi-cytokine cytometric bead array (CBA)

PBMCs were stimulated with an epitope peptide or a peptide mix (each peptide at a final concentration of 1 µg/ml) at 300,000 cells/well of a 96-well U-plate. Culture supernatant was harvested after 72 hours of incubation, and the concentration of IFN-γ, TNF-α and IL-2 was simultaneously determined using CBA (BD Biosciences) and LSRII flow cytometer. In selected patients, concentration of granzyme B in culture supernatants was also determined using CBA.

### Secondary nested RT-PCR for detection of occult HCV infection

To detect occult HCV infection, secondary nested RT-PCR of the 5′-untranslated region of HCV RNA was performed as described elsewhere [32]–[34]. Total RNA was extracted from PBMCs using the RNeasy mini kit (Qiagen, Valencia, CA) with on-column deoxyribonuclease digestion. For cDNA synthesis, 200 to 400 ng RNA was reverse transcribed with ReverTraAce-α kit (Toyobo, Osaka, Japan) using an HCV RNA-specific primer. These cDNA samples served as templates in the direct round of PCR amplification (primer pairs used: 5′-CTG TGA GGA ACT ACT GTC TTC and 5′-GCG GTT GGT GTT ACG TTT), and the nested round reaction (primer pairs used: 5′-GCA GAA AGC GTC TAG CCA TGG C and 5′-CTG CAA GCA CCC TAT CAG GCA GT) was performed with the products of the first round PCR.

For positive cases in secondary nested RT-PCR of HCV RNA, genotypes of HCV were determined as follows. Briefly, the final PCR product of 243 bp was purified and cloned into the TOPO TA cloning vector (Invitrogen, Carlsbad, CA), and genotype was ascertained by automatically sequencing five clones from each patient.

## Results

### Presence of HCV-specific T cell responses in seronegative, aviremic patients

**Figure 1:**
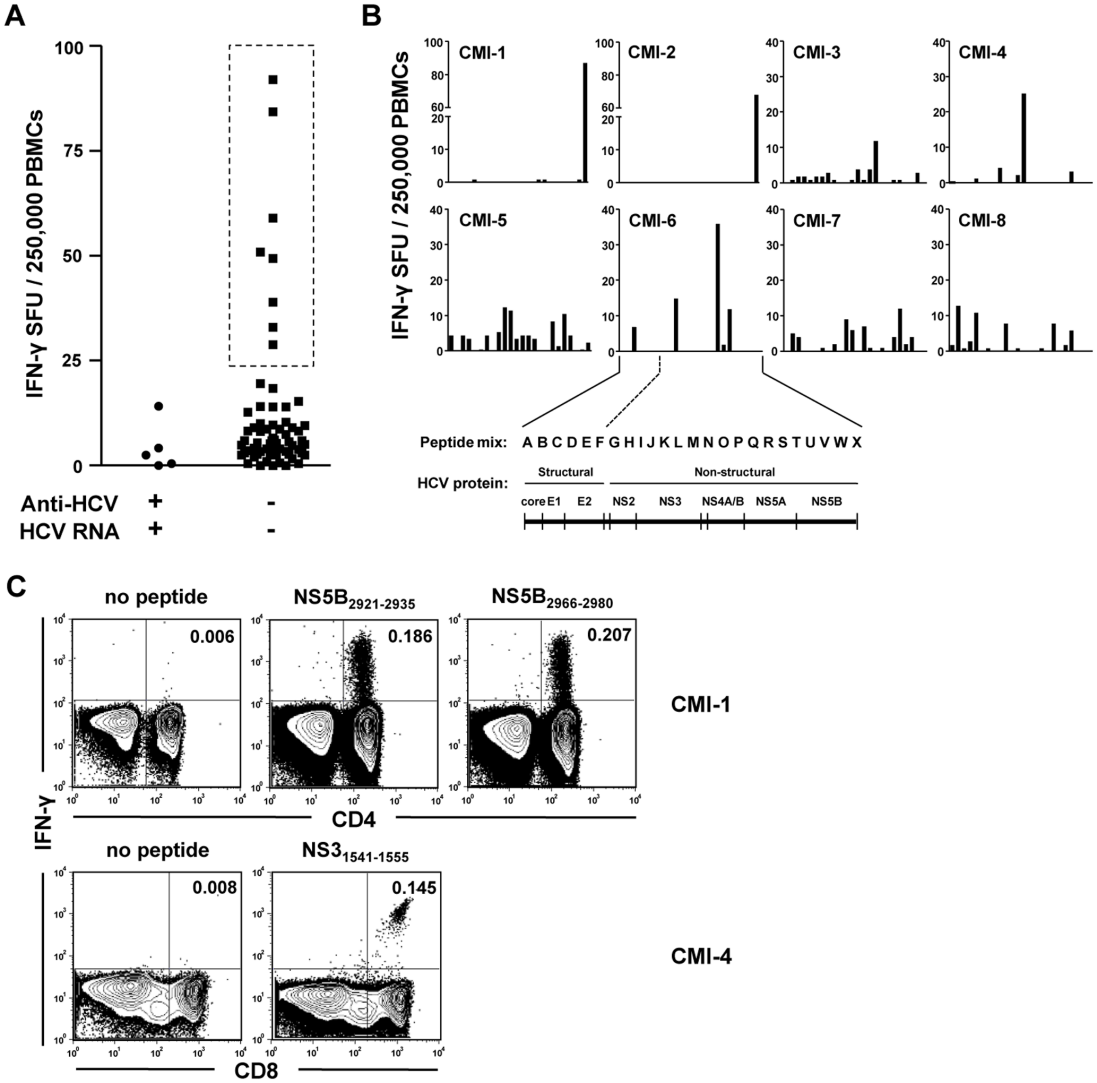
HCV-specific T cell responses in seronegative, aviremic hemodialysis patients. **(A)** Direct *ex vivo* IFN-γ ELISpot assay was performed to examine HCV-specific T cell responses in PBMCs of 76 hemodialysis patients. Five patients were seropositive with viremia (left side), and 71 patients were seronegative and aviremic (right side). PBMCs were stimulated with the 49 matrix mixes of 600 overlapping peptides covering the complete HCV polyprotein sequence (Materials and Methods and Figure S1A). The sum of A-X mixes and the sum of AA-YY mixes were calculated respectively, and their average value was presented for total HCV-specific T cell responses in each patient. Box with dashed lines denotes seronegative, aviremic patients with obvious T cell responses. **(B)** In the eight seronegative, aviremic patients with obvious T cell responses (CMI-1 to CMI-8), the results of IFN-γ ELISpot assay were presented by each peptide mix (A to X). Relationship between peptide mixes and HCV proteins are also displayed. **(C)** IFN-γ ICS was performed to identify the T cell subset responding to HCV epitopes. PBMCs were stimulated with epitope peptides identified in the IFN-γ ELISpot assay in the each patient. FACS dot plots are representative data from two patients, CMI-1 and CMI-4.

Among 77 hemodialysis patients with chronic renal disease, one patient was seropositive without viremia, and five patients were seropositive with viremia. The other 71 patients were seronegative and aviremic, meaning they exhibited no clinical evidence of current or past HCV infection. There were no seronegative patients with viremia. HCV-specific T cell responses were evaluated by direct *ex vivo* IFN-γ ELISpot assay of PBMCs in all patients except the seropositive, aviremic patient. For comprehensive analysis, we stimulated PBMCs with 49 matrixed mixes of 600 overlapping peptides covering the complete HCV polyprotein sequence (Figure S1A). All of the viremic patients and a majority of seronegative, aviremic patients exhibited insignificant levels of HCV-specific T cell responses (Figure 1A). In contrast, eight (11.3%) of the seronegative, aviremic patients displayed obvious T cell responses against HCV (Figure 1A). This was an unexpected result as these HCV-specific T cells were detected in patients without evidence of current or past infection. Figure 1B shows results of the IFN-γ ELISpot assay in response to each peptide mix.

Conducting the IFN-γ ELISpot assay with a matrixed mix of overlapping peptides enabled us to identify T cell epitopes within the HCV polyprotein for each patient (Figure S1B and Table 1). T cell responses to the identified epitopes were confirmed by IFN-γ ICS (Figure 1C). We also identified which T cell subset, CD4^+^ or CD8^+^, was responsible for the HCV-specific IFN-γ production in each patient (Figure 1C and Table 1).

**Table 1:** Seronegative, aviremic hemodialysis patients with HCV-specific T cell responses.

Patient	IFN-γ SFU per 250,000 PBMCs	Responding T cells	Epitope peptides
CMI-1	92.0	CD4	NS5B_2921–2935_, NS5B_2966–2980_
		CD8	N.D.*
CMI-2	39.0	CD4	NS5B_2921–2935_, NS5B_2966–2980_
CMI-3	28.8	N.D.	NS3_1506–1520_, NS3_1521–1535_, NS4B_1881–1895_, NS4B_1896–1910_
CMI-4	33.0	CD8	NS3_1541–1555_
CMI-5	84.3	N.D.	core_35–43_, NS5B_2426–2440_
CMI-6	49.3	CD4	NS3_1131–1145_, NS5A_2006–2020_
CMI-7	59.0	CD4	multi-specific
CMI-8	50.9	CD4	multi-specific

* N.D., not determined.

### Cytokine profiles of HCV-specific T cells in seronegative, aviremic patients

**Figure 2:**
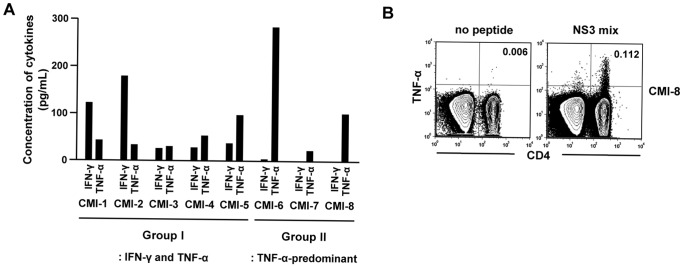
Cytokine profile of HCV-specific T cells in seronegative, aviremic hemodialysis patients. **(A)** After stimulating PBMCs with an epitope peptide or a peptide mix for 3 days, culture supernatant was harvested, and the concentrations of IFN-γ and TNF-α determined in the culture supernatant by CBA. There are two groups of patients in terms of cytokine profile: group I, patients with IFN-γ and TNF-α responses; group II, patients with TNF-α-predominant responses. **(B)** TNF-α ICS was performed in the patients of group II. FACS dot plots are representative data from two patients, CMI-7 and CMI-8.

We further characterized the HCV-specific T cell responses of the eight seronegative, aviremic patients by examining their cytokine profiles. PBMCs were stimulated with an epitope peptide or a peptide mix, and production of both IFN-γ and TNF-α were investigated in culture supernatants. Intriguingly, five patients (CMI-1 to CMI-5, group I) produced both IFN-γ and TNF-α following HCV-specific stimulation, whereas three patients (CMI-6 to CMI-8, group II) produced only TNF-α (Figure 2A). IL-2 production was also assayed and scarcely detected with exception of one patient (CMI-5, data not shown). In the patients of group II, TNF-α production by HCV-specific T cells was confirmed by ICS (Figure 2B). Thus, HCV-specific cytokine profiles distinguish two unique groups of seronegative, aviremic patients with HCV-specific cellular immune responses.

### Two distinct patterns of T cell polyfunctionality in seronegative, aviremic patients

**Figure 3:**
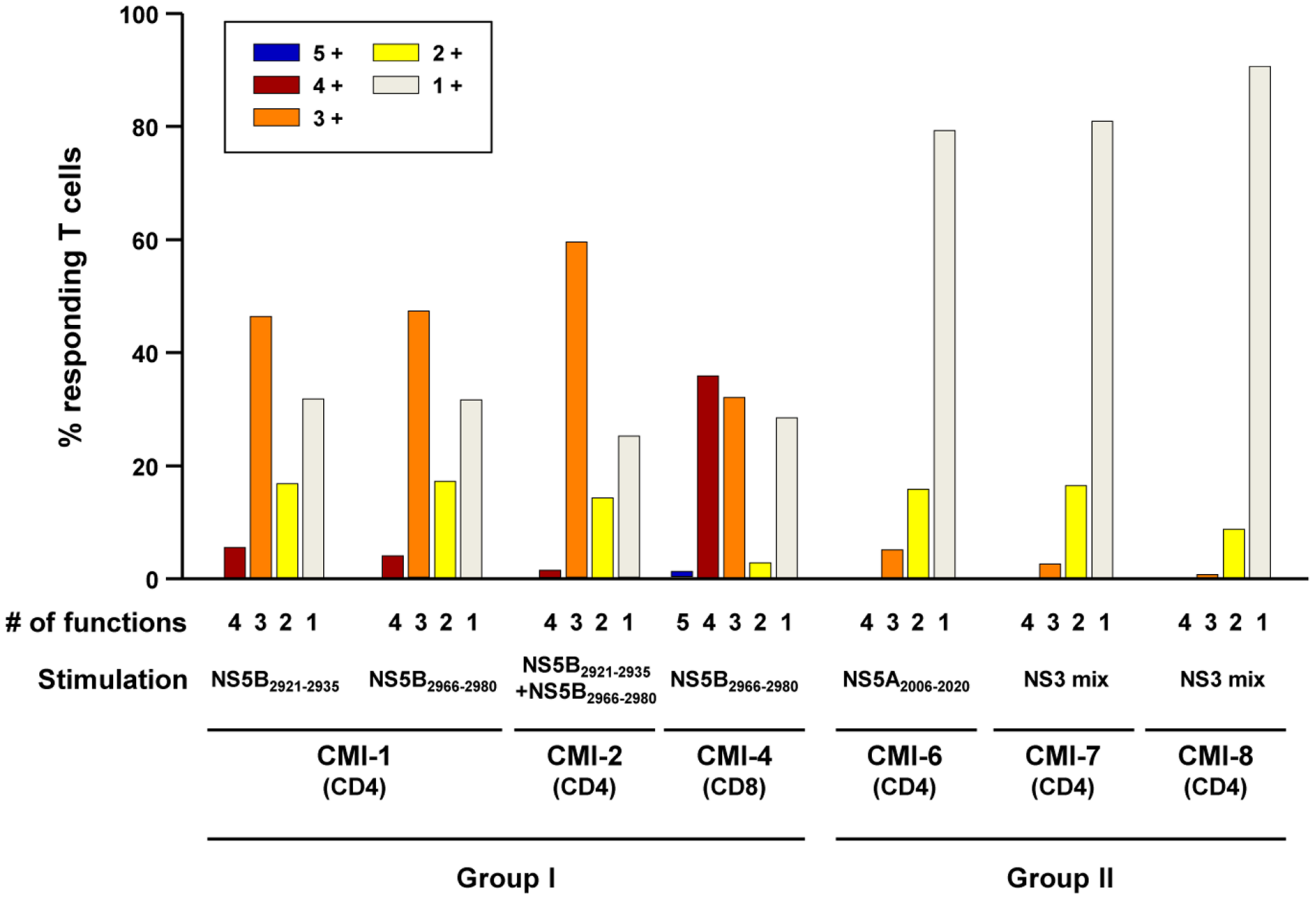
Polyfunctionality of HCV-specific T cells in seronegative, aviremic hemodialysis patients. PBMCs were stimulated with an epitope peptide or a peptide mix, and production of IFN-γ, TNF-α, IL-2, and MIP-1β was simultaneously assessed in multi-cytokine ICS in order to evaluate polyfunctionality of HCV-specific CD4^+^ T cells. In each patient, the fraction of T cells positive for a given number of functions is presented as bar graphs. In the patient with CD8^+^ T cell responses (CMI-4), cytotoxic degranulation function was also evaluated by CD107a staining in addition to multi-cytokine ICS for IFN-γ, TNF-α, IL-2, and MIP-1β.

**Figure 4:**
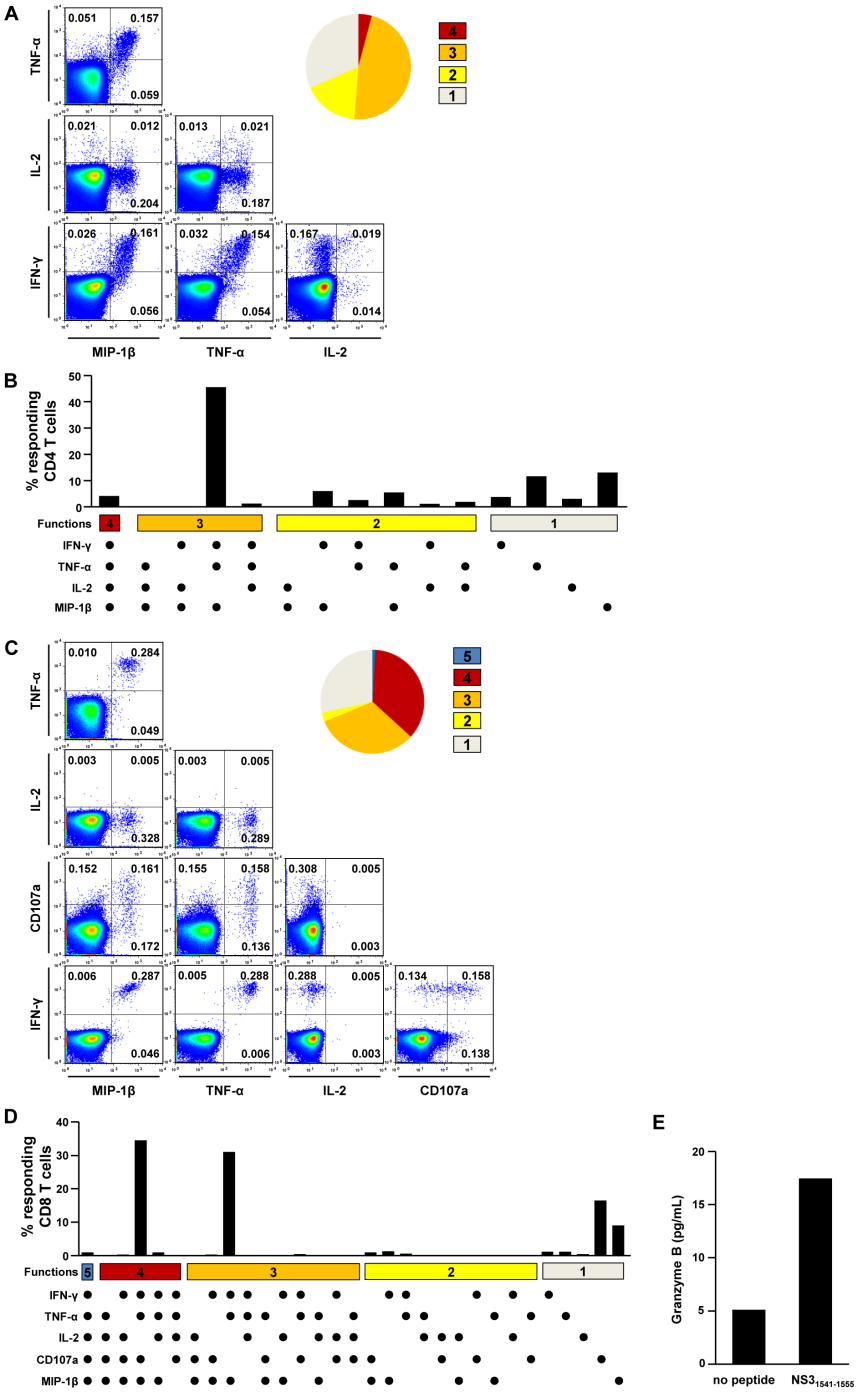
Polyfunctionality assay of HCV-specific T cells in the patients of group I. Representative examples of polyfunctionality assays of CD4^+^ T cells in CMI-1 (A and B) and of CD8^+^ T cells in CMI-4 (C and D) are presented. In CMI-1, PBMCs were stimulated with NS5B_2966–2980_ epitope peptide, and IFN-γ, TNF-α, IL-2, and MIP-1β production was assessed (A and B). In CMI-4, PBMCs were stimulated with NS3_1541–1555_ epitope peptide, and CD107a, IFN-γ, TNF-α, IL-2, and MIP-1β production was assessed (C and D). In the culture supernatant of NS3_1541–1555_ peptide-stimulated PBMCs, concentration of granzyme B was determined by CBA (E). The data are presented by FACS dot plots and the pie graphs show the fraction of T cells positive for a given number of functions (A and C). Detailed analysis of polyfunctionality is presented with every possible combination of functions (B and D).

**Figure 5:**
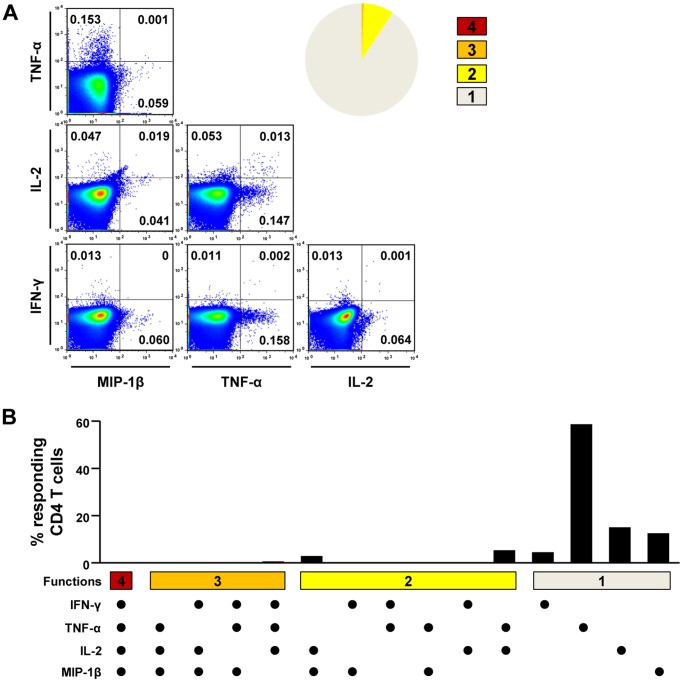
Polyfunctionality assay of HCV-specific T cells in the patients of group II. Representative example of polyfunctionality assays of CD4^+^ T cells in CMI-8 is presented. PBMCs were stimulated with NS3 peptide mix, and IFN-γ, TNF-α, IL-2, and MIP-1β production were assessed. The data are presented by FACS dot plots and the pie graph shows the fraction of T cells positive for a given number of functions (A). Detailed analysis of polyfunctionality is presented with every possible combination of functions (B).

Next, we analyzed T cell polyfunctionality by multi-cytokine ICS to further examine the functional diversity of HCV-specific T cells. In the multi-cytokine ICS of CD4^+^ T cells, IFN-γ, TNF-α, IL-2, and MIP-1β were simultaneously assessed. In the patients of group I, HCV-specific CD4^+^ T cells were proven to be polyfunctional, and more than half of HCV-specific CD4^+^ T cells produced at least three cytokines (group I in Figure 3). In CMI-1, for example, 45.6% of NS5B_2966–2980_-specific CD4^+^ T cells produced IFN-γ, TNF-α, and MIP-1β simultaneously (Figure 4A and 4B). A similar pattern of polyfunctionality was observed against another epitope, NS5B_2921–2935_, in the same patient (Figure S2A and S2B). In the patient with a CD8^+^ T cell response (CMI-4), cytotoxic function was also evaluated in a polyfunctionality assay by the addition of CD107a staining. As a result, more than 60% of HCV-specific CD8^+^ T cells were at least triple-positive, and a major population was quadruple-positive for IFN-γ, TNF-α, MIP-1β, and CD107a (Figure 4C and 4D). In addition, HCV-specific CD8^+^ T cells secreted granzyme B following epitope peptide stimulation (Figure 4E). In contrast, HCV-specific T cells from group II were not polyfunctional (Figure 3). When HCV-specific CD4^+^ T cells were analyzed, 80–90% produced only a single cytokine, TNF-α (Figure 5A and 5B). These data demonstrate that there are two distinct groups, polyfunctional responders and TNF-α-predominant responders, in seronegative, aviremic patients who have HCV-specific memory T cells.

### Neither occult HCV infection nor heterologous immunity is a cause of the T cell responses seen in seronegative, aviremic patients

Recently, occult HCV infection has been demonstrated in persons negative for HCV RNA by clinical-grade RT-PCR [14]. It was reported not only in patients with cryptogenic liver disease [35] but also in hemodialysis patients [36]. Since a previous study reported HCV-specific T cell responses in occult HCV infection [15], we attempted to detect occult HCV infection in seronegative, aviremic patients with HCV-specific cellular immune responses. We performed secondary nested RT-PCR of PBMC samples in all of the hemodialysis patients and obtained a positive result in three seronegative, aviremic patients as well as in all five of the patients positive for HCV RNA by clinical-grade RT-PCR (COBAS TaqMan HCV assay, Roche Diagnostics) (Table 2). However, the three patients with occult HCV infection were not among the eight patients who displayed HCV-specific T cell responses. All eight of the patients with HCV-specific T cell responses showed no sign of occult HCV infection by nested PCR. Thus, occult HCV infection was not a cause of the HCV-specific T cell responses in seronegative, aviremic patients.

**Table 2:** Hemodialysis patients with secondary nested RT-PCR (+).

	Patient	COBAS TaqMan HCV RNA, IU/mL	Nested RT-PCR HCV RNA	Genotype
Seropositive	HepC-1	5.8×10^4^	+	1b
	HepC-2	1.0×10^6^	+	1b
	HepC-3	4.6×10^5^	+	2a
	HepC-4	3.7×10^3^	+	1b
	HepC-5	8.1×10^2^	+	N.D.*
Seronegative	Occult-1	-	+	2a
	Occult-2	-	+	2a
	Occult-3	-	+	N.D.

* N.D., not determined.

Next, heterologous T cell immunity by a cross-reactive epitope was considered as a possible cause of the T cell responses seen in seronegative, aviremic patients. As shown in Table 1, however, T cells from a single patient were specific for multiple HCV epitopes. At least two epitopes were recognized by T cells from each patient, with exception of one, CMI-4 (Table 1). This finding suggested prior T cell priming by *bona fide* HCV proteins, and not by cross-reactive epitopes from other pathogens. In addition, the identified epitope peptides did not show any significant homology in amino acid sequence with peptides from other known pathogens by NCBI database searches. Of interest, T cell epitopes were found not only in structural proteins but also in non-structural (NS) proteins (Figure 1B and Table 1). T cell responses to HCV NS proteins are evidence of *de novo* synthesis of viral proteins in the host. Therefore, we concluded that the HCV-specific T cell responses observed in seronegative, aviremic patients resulted from prior exposure to HCV, even though there was no serological or clinical-grade RT-PCR evidence of current or past HCV infection.

### Comparison of different groups of patients in clinical aspects

In the present study, we identified different groups of hemodialysis patients according to HCV infection and immune status. There were chronic HCV-infected group (seropositive and viremic in clinical-grade RT-PCR) and occult HCV-infected group (seronegative and aviremic, but positive in nested RT-PCR). In addition, there were polyfunctional T cell responder group, TNF-α-predominant T cell responder group and non-responder group in seronegative, aviremic patients. We compared the different groups of patients in terms of demographic and clinical information, including duration of hemodialysis and liver enzymes (Table S1). However, there was no significant difference among groups of patients in demographic and clinical aspects.

## Discussion

HCV-specific cellular immune responses have been demonstrated in seronegative, aviremic persons with no evidence of current or past HCV infection [7]–[13]. This phenomenon was reported in HCV high-risk groups such as IDUs, residents of HCV-endemic areas, healthy family members of HCV-infected patients, and healthcare workers [7]–[13]. Virus-specific T cell responses generated in the absence of apparent infection have also been reported with other viruses. In HIV infection, a portion of seronegative, aviremic persons in the frequent exposure high-risk group displayed strong T cell responses against HIV [37]–[39]. In the present study, we detected HCV-specific T cell responses in 11.3% of seronegative, aviremic hemodialysis patients by direct *ex vivo* IFN-γ ELISpot assays (Figure 1A). The presence of HCV-specific T cell responses in seronegative, aviremic persons has several possible explanations, including occult HCV infection with extremely low-level viral replication [14], [15], and heterologous T cell immunity by a cross-reactive epitope [16]–[18]. However, we showed that occult HCV infection was not a cause of the T cell responses in seronegative, aviremic patients in this study (Table 2). In addition, memory T cells against multiple HCV epitopes in a single patient (Figure 1B and Table 1) suggest that the T cells were primed by *bona fide* HCV proteins, not by cross-reactive epitopes from other pathogens, which is a key element of heterologous immunity. NCBI database searches also suggest that heterologous immunity is not the cause of the HCV-specific T cell responses seen in our study, because the identified HCV epitope peptides did not exhibit significant homology with peptides derived from other pathogens. Similarity in the three dimensional structure, rather than the primary sequence, also needs to be considered in cross-reactivity of HCV epitopes [40].

Our data indicate that HCV-specific memory T cells in seronegative, aviremic patients arose from previous exposure to HCV and subclinical transient viral replication without seroconversion. Previous HCV replication in these patients is evidenced by T cell specificity for epitopes derived from HCV NS proteins (Figure 1B and Table 1), which would require *de novo* synthesis of viral proteins in the host. In a previous cohort study, HCV-specific cellular immune responses were detected in self-limited acute HCV infection without seroconversion [19]. In addition, another study directly demonstrated that exposure to low doses of HCV led to HCV-specific T cell responses in chimpanzees, with transient viral replication [20]. The same study found that HCV challenge with 1-10 RNA (+) inoculums resulted in priming of HCV-specific T cells without sustained viremia or seroconversion [20]. It remains possible that in our patients, anti-HCV antibody was generated during an acute infection and has over the course of many years disappeared from circulation. In fact, a previous study demonstrated that anti-HCV disappeared 20 years after recovery from acute HCV infection while cellular immune responses persisted [21]. Clinical implications of HCV-specific T cell response in seronegative, aviremic persons will require further investigation. In addition, phenotypic characteristics of HCV-specific T cells needs to be analyzed in further studies, including memory markers, activation markers and exhaustion markers.

In the present study, we showed two distinct patterns of T cell polyfunctionality in seronegative, aviremic patients (Figures 3, 4, and 5). HCV-specific memory T cells were highly polyfunctional in some patients (group I in Figure 3), whereas they were TNF-α-predominant in the others (group II in Figure 3). Recently, T cell polyfunctionality has been investigated in HIV infection and vaccination studies [27]–[29] and it was found that polyfunctional HIV-specific CD8^+^ T cells were maintained in HIV long-term nonprogressors [28]. Furthermore, in a Leishmania vaccination study, the degree of Th1 cell polyfunctionality was shown to correlate with vaccine efficacy [27]. In addition, the profound efficacy of the smallpox vaccine has been attributed to polyfunctionality of virus-specific CD8^+^ T cells. Taken together, polyfunctional HCV-specific memory T cells in seronegative, aviremic patients would be expected to protect against subsequent exposure to HCV. Intriguingly, a very recent study demonstrated that polyfunctional HCV-specific T cells were associated with vaccine-induced control of HCV [41].

In patients with a TNF-α-predominant response, HCV-specific T cells might not provide antiviral protection upon subsequent HCV exposure. We can infer the absence of a protective role for TNF-α-single-positive T cells from a recent study of tuberculosis. In this study, TNF-α-single-positive *Mycobacterium tuberculosis*-specific CD4^+^ T cells were preferentially detected in active tuberculosis disease rather than in latent infection [42]. It is possible that TNF-α-single-positive T cell responses might serve solely as evidence of T cell priming by exposure to pathogen.

In the present study, we suggest that HCV-specific memory T cells of seronegative, aviremic patients might result from transient viral replication without seroconversion or, alternately, from disappearance of anti-HCV antibody long after prior HCV infection. If these assumptions prove true, it would suggest that past HCV exposure may be better assessed by HCV-specific T cell responses than by presence of anti-HCV antibody. In fact, HCV-specific T cell assays could confirm HCV exposure in indeterminate blood donors [43]–[45]. However, T cell assays for IFN-γ responsiveness might underestimate HCV exposure since there are seronegative, aviremic patients who demonstrate a TNF-α-predominant response. Thus, assays specific for a T cell TNF-α response might prove superior to assays examining IFN-γ production.

In summary, we show that HCV-specific T cell responses in seronegative, aviremic hemodialysis patients might result from a prior, transient viral replication without seroconversion or from disappearance of anti-HCV long after acute HCV infection has resolved, but not from current occult HCV infection. We also demonstrate that HCV-specific T cell responders in seronegative, aviremic hemodialysis patients are classified into two distinct groups, polyfunctional responders and TNF-α-predominant responders. Determining the significance of these responses with respect to immunology and vaccinology will require further investigation. In particular, detailed characterization of HCV-specific T cell polyfunctionality might provide a basis for understanding of protective immunity against HCV and a strategy for the development of an HCV vaccine.

## Supporting Information

Figure S1 **Matrix array of HCV overlapping peptides and identification of epitope candidates.**
(TIF)

Figure S2 **Polyfunctionality assay of HCV-specific T cells in CMI-1 with the second epitope peptide.**
(TIF)

Table S1 **Demographic and clinical characteristics of major groups in hemodialysis patients.**
(DOC)
