# Supplementary figures and images for: Two Distinct Functional Patterns of Hepatitis C Virus (HCV)-Specific T Cell Responses in Seronegative, Aviremic Patients
Source: PLoS One. 2013 Apr 30;8(4):e62319. doi: 10.1371/journal.pone.0062319 (PMC3640053; doi:10.1371/journal.pone.0062319)

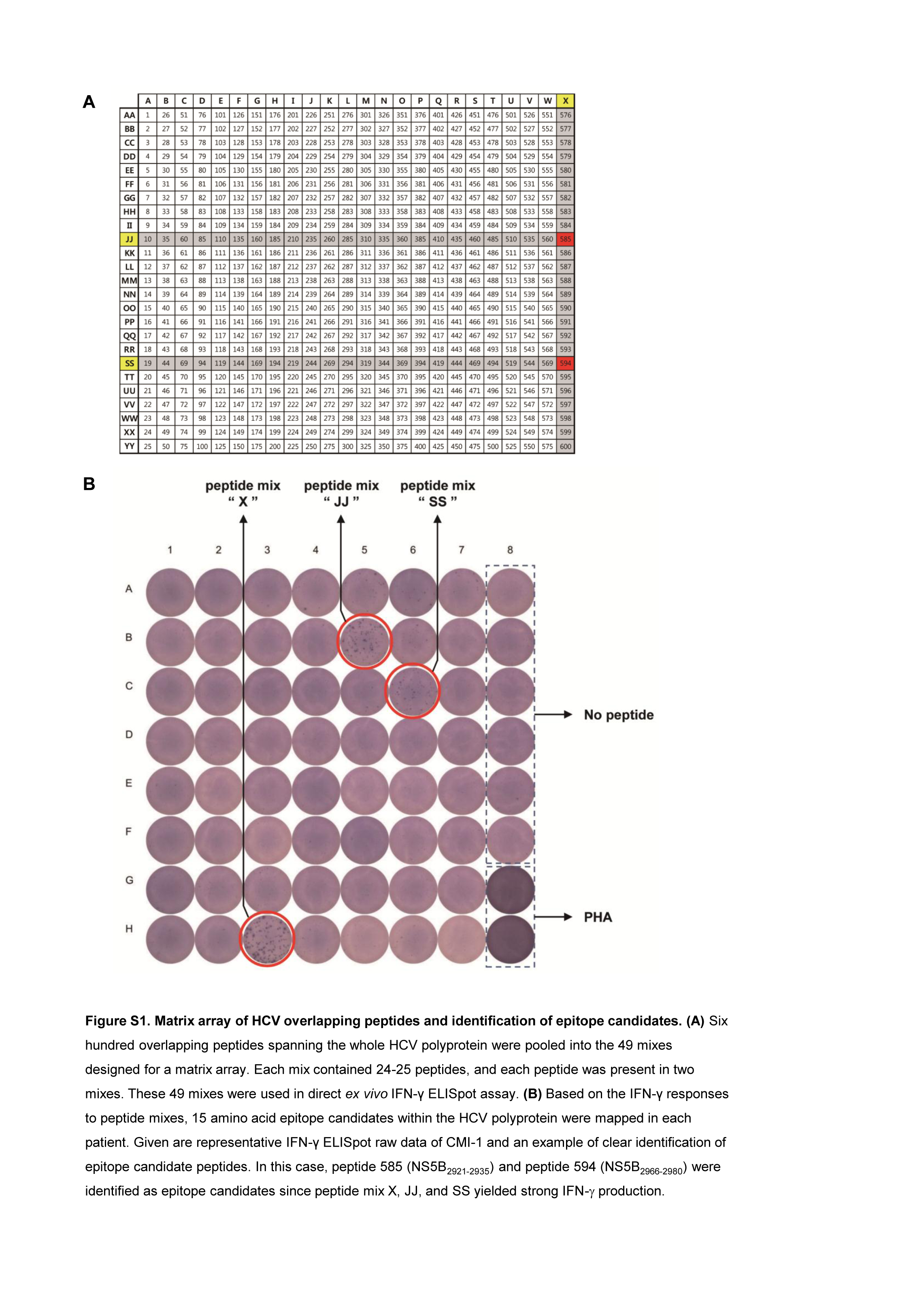

Supplement: Figure S1 — Matrix array of HCV overlapping peptides and identification of epitope candidates. (TIF) [file pone.0062319.s001.tif]

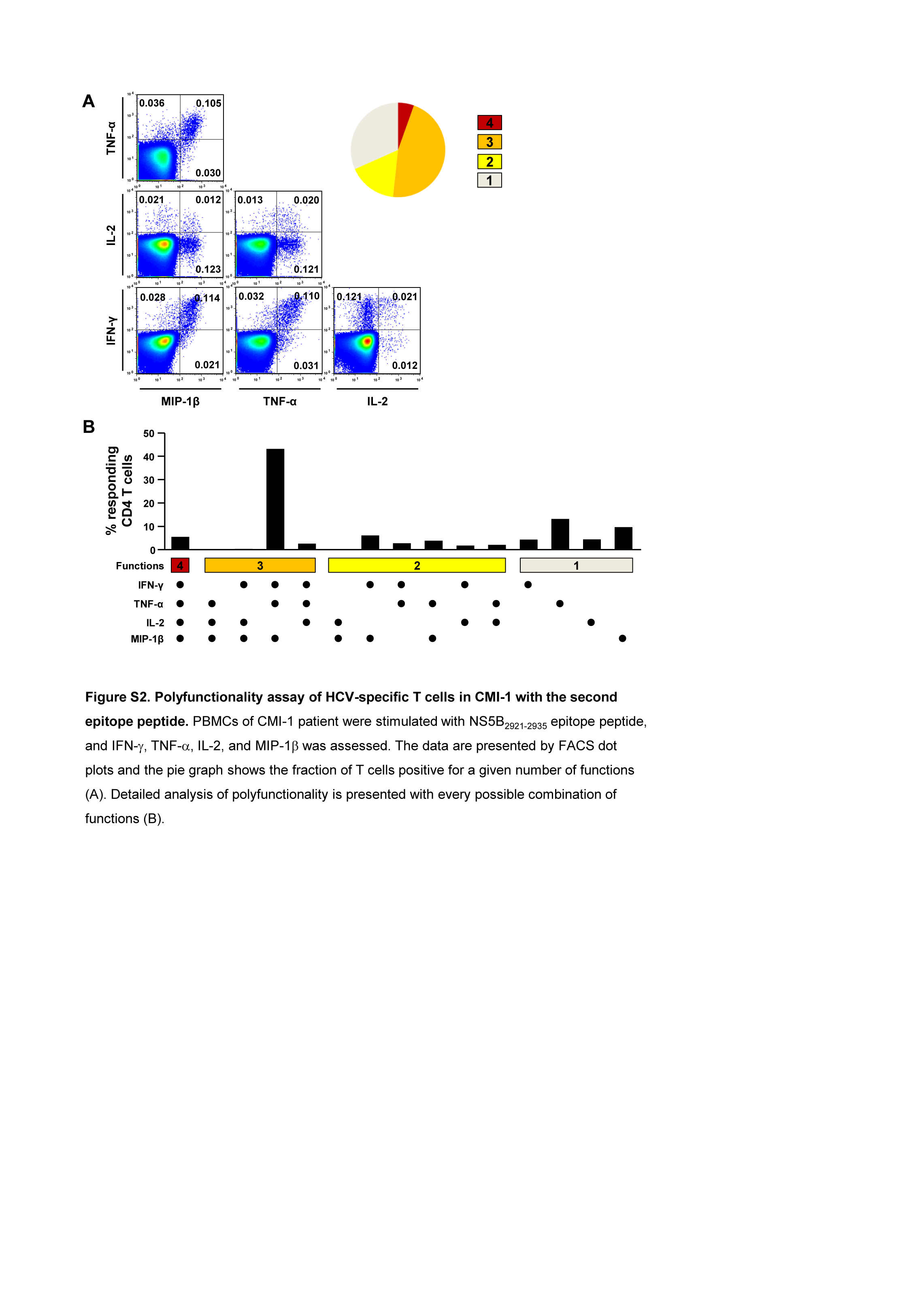

Supplement: Figure S2 — Polyfunctionality assay of HCV-specific T cells in CMI-1 with the second epitope peptide. (TIF) [file pone.0062319.s002.tif]
