# Supplementary material for: Two Distinct Functional Patterns of Hepatitis C Virus (HCV)-Specific T Cell Responses in Seronegative, Aviremic Patients
Source: PLoS One. 2013 Apr 30;8(4):e62319. doi: 10.1371/journal.pone.0062319 (PMC3640053; doi:10.1371/journal.pone.0062319)
Supplement: Table S1 — Demographic and clinical characteristics of major groups in hemodialysis patients. (DOC) [file pone.0062319.s003.doc]

**Table S1.** Demographic and clinical characteristics of major groups in hemodialysis patients.

| Group | Patient | Gender | Age, yr | Duration of HD, mo | Serum ALT, IU/L | Serum AST, IU/L | IFN-γ SFU per 250,000 PBMCs |
| --- | --- | --- | --- | --- | --- | --- | --- |
| Chronic hepatitis C |  |  |  |  |  |  |  |
|  | HepC-1 | F | 66 | 297.0 | 20 | 17 | 0.5 |
|  | HepC-2 | M | 61 | 70.3 | 14 | 32 | 4.2 |
|  | HepC-3 | F | 62 | 42.2 | 15 | 21 | 2.5 |
|  | HepC-4 | M | 48 | 282.5 | 22 | 19 | 0.0 |
|  | HepC-5 | F | 67 | 9.0 | 9 | 15 | 14.2 |
| Occult HCV infection |  |  |  |  |  |  |  |
|  | Occult-1 | F | 52 | 8.3 | 14 | 20 | 2.7 |
|  | Occult-2 | M | 62 | 48.4 | 16 | 18 | 9.0 |
|  | Occult-3 | F | 64 | 6.3 | 12 | 15 | 12.8 |
| Polyfunctional response |  |  |  |  |  |  |  |
|  | CMI-1 | F | 52 | 52.9 | 12 | 10 | 92.0 |
|  | CMI-2 | M | 63 | 75.6 | 99 | 22 | 39.0 |
|  | CMI-3 | F | 71 | 26.9 | 29 | 31 | 28.8 |
|  | CMI-4 | M | 54 | 30.5 | 22 | 22 | 33.0 |
|  | CMI-5 | F | 58 | 101.1 | 25 | 14 | 84.3 |
| TNF-α-predominant response |  |  |  |  |  |  |  |
|  | CMI-6 | M | 70 | 26.5 | 24 | 27 | 49.3 |
|  | CMI-7 | M | 27 | 116.2 | 17 | 17 | 59.0 |
|  | CMI-8 | F | 31 | 19.3 | 34 | 22 | 50.9 |
